# Purification and characterization of endo β-1,4-d-glucanase from *Trichoderma harzianum* strain HZN11 and its application in production of bioethanol from sweet sorghum bagasse

**DOI:** 10.1007/s13205-016-0421-y

**Published:** 2016-04-12

**Authors:** Zabin K. Bagewadi, Sikandar I. Mulla, Harichandra Z. Ninnekar

**Affiliations:** Department of Biochemistry, Karnatak University, Dharwad, 580 003 Karnataka India

**Keywords:** Endo β-1,4-d-glucanase, *Trichoderma harzianum* strain HZN11, Purification, Characterization, Enzymatic hydrolysis, Bioethanol

## Abstract

An acidophilic-solvent-thermostable endo β-1,4-d-glucanase produced from a potential *Trichoderma harzianum* strain HZN11 was purified to homogeneity by DEAE-Sepharose and Sephadex G-100 chromatography with 33.12 fold purification with specific activity of 66.25 U/mg and molecular mass of ~55 kDa. The optimum temperature and pH were 60 °C and 5.5 retaining 76 and 85 % of activity after 3 h, respectively. It showed stability between pH 4.5–6.0 and temperature between 50–70 °C indicating thermostability. Endo β-1,4-d-glucanase was activated by Ca^2+^ and Mg^2+^ but inhibited by Hg^2+^, Pb^2+^ and Cd^2+^. The effect of thiol reagents, metal chelators, oxidizing agents and surfactants on enzyme activity has been studied. Purified endo β-1,4-d-glucanase exhibited highest specificity towards carboxymethyl cellulose. Kinetic analysis showed the *K*_m_, *V*_max_ and *K*_i_ (cellobiose inhibitor) of 2.5 mg/mL, 83.75 U/mg and 0.066 M, respectively. The storage stability of purified endo β-1,4-d-glucanase showed a loss of mere 13 % over a period of 60 days. The hydrolysis efficiency of purified endo β-1,4-d-glucanase mixed with cocktail was demonstrated over commercial enzyme. Optimized enzymatic hydrolysis of sweet sorghum and sugarcane bagasse released 5.2 g/g (36 h) and 6.8 g/g (48 h) of reducing sugars, respectively. Separate hydrolysis and fermentation of sweet sorghum bagasse yielded 4.3 g/L bioethanol (16 h) confirmed by gas chromatography–mass spectrometry (GC–MS). Morphological and structural changes were assessed by scanning electron microscopy (SEM) and Fourier transform infrared spectroscopy. Elemental analysis was carried out by SEM equipped with energy dispersive X-ray technique. These unique properties prove the potentiality of enzyme for biomass conversion to biofuel and other industrial applications.

**Electronic supplementary material:**

The online version of this article (doi:10.1007/s13205-016-0421-y) contains supplementary material, which is available to authorized users.

## Introduction

In the current scenario, the major concerns are towards the diminishing of fossil fuels which have forced the energy industries and researchers to develop alternatives to the existing fuels (Bentsen and Felby 2012). One of the attractive sustainable substitutes is the microbial production of bioethanol from lignocellulosic wastes as it is cost-effective and renewable (Ren et al. 2009). Plant biomass constitute of cellulose which is the major organic polysaccharide found in the biosphere (Bhat and Bhat 1997) and is renewable. Biodegradation of plant based biomass requires cellulose and hemicellulose saccharifying enzymes. For example, cellulases participate in saccharification of biomass for bioethanol production (Dhillon et al. 2011), by mainly acting on β-1,4-glycosidic bonds of cellulose. Cellulolytic enzymes have been classified as: endoglucanase (endo-1,4-d-glucanase, EG), cellobiohydrolase (exo-1,4-d-glucanase, CBH) and glucosidase (1,4-d-glucosidase, BG) (Saha 2004), which have been shown to act synergistically for effective degradation (Lynd et al. 2002) whereas xylanases (1,4-β-d-xylanohydrolase) hydrolyze xylan, a major component of hemicellulose (Zhang et al. 2011). The fungal endoglucanases finds its applications in biomass bioconversions, pulp and paper, textile, detergents, starch processing, grain alcohol fermentation, brewery, wine making, extraction of fruit and vegetable juices (Karmakar and Ray 2011; Kuhad et al. 2011). These applications certainly require endoglucanases with industrial attributes like thermostability, stability at varying pH, substrate specificities (Bhat 2000), solvent tolerant, detergent compatibility, chemical stability, etc. Solid state fermentation (SSF) for cellulase production is an advantageous process as it reduces the capital investment with easy operating conditions (Pandey et al. 1999). Cellulose saccharification can be carried out by separate hydrolysis and fermentation (SHF) process with an ease of optimizing the enzymatic hydrolysis conditions (Zhu et al. 2012) for ethanol production. Ethanol quantification can be achieved by employing methods like GC–MS. Desirable for better specificity, few mass spectrometric (MS) methods for ethanol analysis have been reported (Tiscione et al. 2011). Insights of molecular level changes and functional groups in the lignocellulosic material at various fermentation steps could be studied by employing FTIR (Adapa et al. 2011; Sim et al. 2012), morphological changes by SEM and substrate elemental analysis by higher throughput techniques like SEM equipped with EDX technique.

Keeping in view the industrial applications of the endo β-1,4-d-glucanase, this study was carried out to purify and characterize a novel endo β-1,4-d-glucanase from *Trichoderma harzianum* strain HZN11. Enzymatic hydrolysis and ethanol fermentation was successfully achieved. Further, sweet sorghum bagasse was molecularly characterized with techniques like FTIR, SEM and SEM/EDX.

## Materials and methods

### Chemicals, substrate and culture

All the chemicals and media components used were procured from HiMedia, Sigma-Aldrich (USA) and Merck (USA). Sweet sorghum stalks were collected from University of agricultural sciences, Dharwad. *Saccharomyces cerevisiae* NCIM 3594 was procured from National Collection of Industrial Microorganisms (NCIM).

### Fungal strain and production of endo β-1,4-d-glucanase

*Trichoderma harzianum* strain HZN11 previously isolated from forest soil was identified based on 18S rDNA sequencing (data was not shown). The nucleotide sequence of the strain was deposited to NCBI (National Center for Biotechnology Information) GenBank with accession number KP050786. The newly isolated *Trichoderma harzianum* strain HZN11 is maintained at the Department of Biochemistry, Karnatak University, Dharwad on potato dextrose agar (PDA) enriched with carboxymethyl cellulose (CMC) at 4 °C. Endo β-1,4-d-glucanase was produced by *Trichoderma harzianum* strain HZN11 in SSF using alkali pretreated sweet sorghum bagasse as substrate. SSF was carried out in 250 mL Erlenmeyer flasks containing 10 g of pretreated substrate in Mandels–Weber medium containing (g/L) urea 0.3; ammonium sulfate 1.4; KH_2_PO_4_ 0.3; CaCl_2_ 0.3; MgSO_4_.7H_2_O 0.3; protease peptone 1.0; lactose 10; and (mg/L) FeSO_4_.7H_2_O 5.0; MnSO_4_.7H_2_O 1.6; ZnSO_4_.7H_2_O 1.4; CoCl_2_ 2; Tween-80 0.1 %; and pH 6 with 70 % moisture content. Sterilized flasks were inoculated with 4 mL spore suspension and incubated at 35 °C under static condition for 7 days. The crude enzyme was extracted with 50 mM sodium acetate buffer, pH 6 with 1:2 solid to liquid ratio under shaking (150 rpm) at 35 °C for 30 min, followed by filtration through muslin cloth. The filtrate was centrifuged at 8000 rpm for 20 min at 4 °C. The clear supernatant was used as crude enzyme for purification.

### Enzyme assay and protein determination

Endo β-1,4-d-glucanase hydrolyzes CMC to produce free carboxymethyl glucose units. Endo β-1,4-d-glucanase activity was estimated using CMC as substrate under standard conditions according to Standard International Union of Pure and Applied Chemistry (IUPAC) method described by Ghose (1987). The reducing sugars released from the reaction were determined according to Miller (1959) by dinitrosalicylic acid (DNS) method. In the above assay, one unit (U) of enzyme was defined as the amount of enzyme that released 1 μmol of the glucose per minute under standard assay conditions (30 min incubation at 50 °C with 50 mM acetate buffer pH 6.0). The concentrations of soluble proteins were estimated according to Lowry et al. (1951) using bovine serum albumin (BSA) as the standard.

### Purification of endo β-1,4-d-glucanase and molecular mass determination

Endo β-1,4-d-glucanase produced from sweet sorghum bagasse by *Trichoderma harzianum* strain HZN11 was subjected for purification. The cell debris was removed by vacuum filtration (Millipore India Ltd.) and the enzyme protein was subjected to fractionation by (NH_4_)_2_SO_4_ (70 % w/v). The precipitate was centrifuged at 8000 rpm for 15 min at 4 °C. Enzyme was resuspended in a minimum volume of 50 mM sodium acetate buffer (pH 6.0) and dialyzed against the same buffer for 6 h at 4 °C and lyophilized. Total proteins and endo β-1,4-d-glucanase activity of partially purified fraction were determined before and after dialysis. Endo β-1,4-d-glucanase was further purified by column chromatography. The lyophilized enzyme was dissolved in 50 mM sodium acetate buffer (pH 6.0). The enzyme protein was loaded onto DEAE-Sepharose column (40 × 2 cm) pre-equilibrated with 50 mM sodium acetate buffer (pH 6.0). The flow rate was maintained at 1 mL/min and eluted with gradient of 0.1–1 M NaCl. The pooled fractions were dialyzed and lyophilized. The fractions were re-dissolved in buffer and loaded onto the Sephadex G-100 column (30 × 2 cm) pre-equilibrated and eluted with 50 mM sodium acetate buffer (pH 6.0) at a flow rate of 0.5 mL/min. The fractions were analyzed for proteins by A_280_ method and endo β-1,4-d-glucanase activities were determined. The resulting concentrated active endo β-1,4-d-glucanase fractions were pooled and used for further characterization (Bakare et al. 2005). The apparent molecular weight of the purified endo β-1,4-d-glucanase was determined by sodium dedocylsulphate polyacrylamide gel electrophoresis (SDS-PAGE) with protein molecular weight ladder [lysozyme (14.3 kDa), β-lactoglobulin (20 kDa), carbonic anhydrase (29 kDa), ovalbumin (43 kDa), bovine serum albumin (66 kDa) and phosphorylase B (97.4 kDa) run along with sample], according to the method described by Laemmli (1970). Protein bands were visualized by staining with coomassie brilliant blue R-250.

### Characterization of purified endo β-1,4-d-glucanase

Optimum pH of the purified endo β-1,4-d-glucanase was determined by incubating the enzyme at different pH ranging from pH 3–11 using 0.1 M buffers of sodium citrate (pH 3–4.5), sodium acetate (pH 5–6), sodium phosphate (pH 6.5–7.5), Tris–HCl (pH 8.0–9.5), and glycine–NaOH (pH 10.0–11.0). The stability of pH was determined at optimum range of pH 5–6 for 3 h and the relative activity was calculated. Optimum temperature was evaluated in range of 20–85 °C and the thermo stability of endo β-1,4-d-glucanase was assessed between optimum temperature ranges of 50–65 °C for 3 h at pH 5.5 by measuring the relative activity. Effect of various metal ions like Co^2**+**^, Zn^2**+**^, Ca^2**+**^, Mg^2**+**^, K^**+**^, Na^**+**^, Cu^2**+**^, Hg^2**+**^, Fe^2**+**^, Pb^2**+**^, Ni^2**+**^, Mn^2**+**^ and Cd^2**+**^ was determined at different concentrations (1–10 mM). Endo β-1,4-d-glucanase relative activity was evaluated in the presence of various additives like dithiothreitol (DTT), β-mercaptoethanol, ethylene diamine tetra acetic acid (EDTA), urea, phenyl methyl sulphonyl fluoride (PMSF), *N*-bromosuccinimide, dimethyl sulfoxide (DMSO), iodoacetamide, *p*-chloromercuribenzoate (*p*-CMB) and 1,10-phenanthroline, at various concentrations (1–10 mM). Relative activities of endo β-1,4-d-glucanase in the presence of various detergents like sodium dodecyl sulfate (SDS), sodium tetraborate, and commercial detergents (Tide, Ariel and Surf Excel), surfactants like tween-20, tween-40, tween-80 and triton X-100 and oxidizing agents like sodium perborate, sodium hypochlorite and hydrogen peroxide at varying concentrations (0.1–1 %) were determined. Endo β-1,4-d-glucanase stability in the presence of various organic solvents like glycerol, ethanol, methanol, acetone, formic acid, propanol, petroleum ether, isopropanol, benzene, cyclohexane, hexane, butanol and toluene at different concentrations (10–30 %) were evaluated (control was 100 %). Substrate specificity of endo β-1,4-d-glucanase was tested against variety of substrates like 1 % microcystalline cellulose, CMC, chitin, cellobiose, starch, filter paper, PNP-α-galactopyranoside, PNP-glucopyranoside, PNP-cellobioside, brichwood xylan and oat spelt xylan. Endo β-1,4-d-glucanase kinetics with CMC concentration range of 2.5–30 mg/mL was studied and kinetic parameters *K*_m_ and *V*_max_ were determined by linear transformations of the Michaelis–Menten model to Lineweaver–Burk. Inhibition of endo β-1,4-d-glucanase with cellobiose was studied at 5 mM and 10 mM and inhibition constant *K*_i_ were determined by Lineweaver–Burk plot. The storage stability of the purified endo β-1,4-d-glucanase was monitored for 60 days.

### Enzymatic hydrolysis and ethanol fermentation

Enzymatic hydrolysis of untreated and alkali pretreated sweet sorghum bagasse and sugarcane bagasse was studied using purified endo β-1,4-d-glucanase (53 U/g) mixed with crude multi-enzyme cocktail (exoglucanase 15 U/g, filter paper activity (FP) 15 U/g, cellobiase 18 U/g, xylanase 1740 U/g and β-glucosidase 13 U/g) produced by *Trichoderma harzianum* strain HZN11 from sweet sorghum bagasse and commercial cellulase from *Trichoderma* sps. (9 FP U/mL) individually. Reaction constituted of 2 % substrate in 50 mM sodium acetate buffer pH 5.5, 0.1 % tween-40 and filter sterilized enzyme in a volume of 30 mL incubated at 40 °C at 150 rpm. The reaction was fortified with 0.005 % sodium azide. Samples were withdrawn, centrifuged at 8000 rpm for 15 min and the clear supernatants were analyzed for reducing sugars according to the method described by Miller (1959). Controls were kept for each reaction with heat-inactivated enzyme. Parameters such as hydrolysis time (12–72 h) and temperature (40–60 °C) for enzymatic hydrolysis of alkali pretreated sweet sorghum bagasse and sugarcane bagasse was optimized. SHF experiments were designed in which hydrolyzates of sweet sorghum bagasse were collected and centrifuged at 8000 rpm for 15 min. The supernatant containing reducing sugars was transferred to serum bottles for fermentation process with pH maintained to 7 by 1 N NaOH. Glucose fermenting yeast, *Saccharomyces cerevisiae* NCIM 3594 was inoculated and incubated at 30 °C for 72 h under shaking at 120 rpm. Aliquots were withdrawn at different time intervals for the estimation of ethanol, reducing sugars and biomass.

### Analytical methods

The SHF aliquot samples were filtered through 0.2 mm membrane filters for the analysis of ethanol by GC–MS (ShimadzuQP2010 Plus) equipped with an autosampler. GC–MS accelerates ethanol analysis with its simultaneous separation and identification. GC–MS was equipped with quadruple mass filter Rtx-5MS capillary column (30 m × 0.25 mm), scan interval 0.5 s and mass range 40–500 *m*/*z*. The column temperature was maintained at 50 °C for 1 min, and then ramped with 20 °C increase per min to a final temperature at 280 °C for 14.5 min and the injector temperature was kept at 250 °C. Helium was used as carrier gas in the gas chromatographer with 1 µL injection volume. The MS was operated at electron ionization energy of 70 eV. The sample was run for 20 min. Absolute ethanol was used as standard.

The morphology and physical property changes of untreated, alkali pretreated and *Trichoderma harzianum* strain HZN11 hydrolyzed sweet sorghum bagasse samples were analyzed by SEM (JOEL-JSM 5600, JAPAN). The lyophilized samples were mounted on aluminum stubs, and sputter-coated with a gold layer. The SEM images were taken at different magnifications. SEM equipped with EDX technique was used for elemental analysis of the substrate.

FTIR (Perkin Elmer, FTIR1760) was used to investigate the structural changes in untreated, alkali pretreated and *Trichoderma harzianum* strain HZN11 hydrolyzed sweet sorghum bagasse samples. Samples were mixed with KBr (potassium bromide) and prepared pellets. The spectra of samples were obtained using 32 scans with the spectra resolution measured as 4 cm^−1^ with a scanning range of 500–4000 cm^−1^.

## Results and discussion

### Purification of endo β-1,4-d-glucanase and molecular mass determination

The endo β-1,4-d-glucanase produced by *Trichoderma harzianum* strain HZN11 was purified to homogeneity by (NH_4_)_2_SO_4_ precipitation, DEAE-Sepharose and Sephadex G-100 chromatography. The enzyme was purified to 33.12 fold with specific activity of 66.25 U/mg protein as shown in Table 1. The elution profiles of DEAE-Sepharose and Sephadex G-100 chromatography are shown in Fig. S1 (Supplementary Information, SI) which did not show any multiple isoforms of enzyme.

**Table 1:** Purification summary of endo β-1,4-d-glucanase from *Trichoderma harzianum* strain HZN11

Purification steps	Total volume (mL)	Total protein (mg)	Total activity (U)	Specific activity (U/mg)	Yield (%)	Fold purification
Crude extract	200	1800	3600	2	100	1
Ammonium sulfate	25	325	1150	3.53	32	1.765
Fractionation (70 %)						
DEAE-Sepharose	8	32.8	456	13.9	12.7	6.95
Sephadex G-100	4	3.2	212	66.25	5.9	33.12

**Fig. 1:**
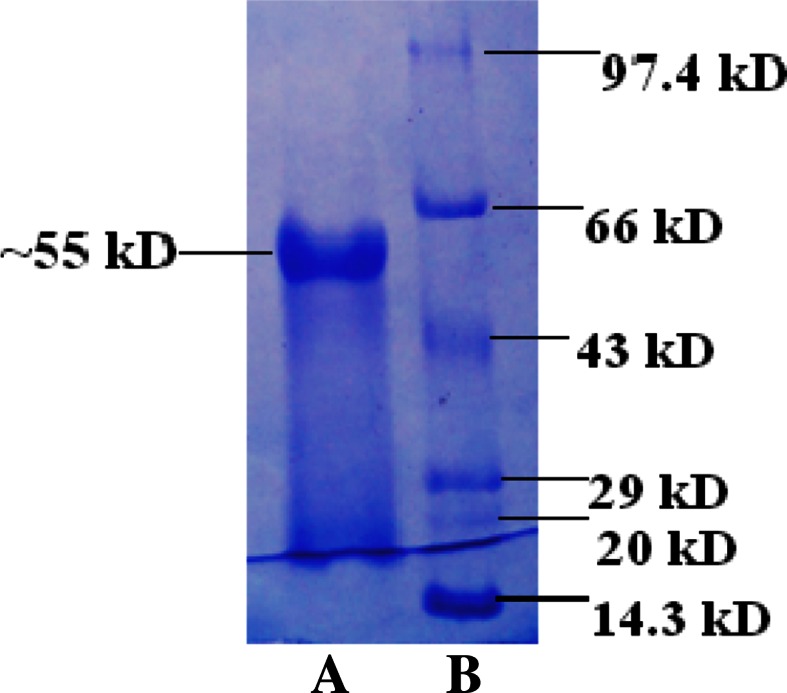
SDS-PAGE with lane **a**: purified endo β-1,4-d-glucanase and lane **b**: Molecular weight markers

The purified endo β-1,4-d-glucanase showed a single protein band on SDS-PAGE with molecular weight ~55 kDa (Fig. 1) and was found to be a monomeric protein from native gel (data not shown). There are reports of varying molecular mass of endo β-1,4-d-glucanase from different organisms, 29 kDa from *Aspergillus niger* AT-3 (Dutt and Kumar 2014) and 62 kDa from *P. betulinus* (Valaskova and Baldrian 2006).

### Characterization of purified endo β-1,4-d-glucanase

#### Effect of pH and temperature

The purified endo β-1,4-d-glucanase was remarkably active in the pH range of 4–6.5 with optimum pH at 5.5 as shown in Fig. S2A (SI). Fungal cellulases have found to be active in acidic pH. The charge distribution on substrate and enzyme is greatly affected by variation in pH intern affecting the mechanism of substrate binding and catalysis (Shah and Madamwar 2005). Enzyme stability at its optimum pH is a required for efficient catalysis. 81, 85 and 77 % of activity was retained at pH 5, 5.5 and 6, respectively, after 3 h (Fig. S2B, SI), thereby anticipating good pH stability. Similarly, effect of temperature was evaluated in the range between 20–85 °C. Endo β-1,4-d-glucanase from *Trichoderma harzianum* strain HZN11 was found to be highly stable between 50–70 °C with optimum activity at 60 °C indicated in Fig. S2C (SI). The relative activity was recorded to be 80, 77, 76 and 66 % at 50, 55, 60 and 65 °C, respectively, after 3 h (Fig. S2D, SI). The results reveal an acidic pH stable and thermostable endo β-1,4-d-glucanase from *Trichoderma harzianum* strain HZN11 which could be employed for enzymatic hydrolysis of lignocellulosic biomass. Thermal stability of an enzyme is an attractive property for significant industrial applications (Asgher and Iqbal 2011). Many investigators reported optimum reaction temperature of 50–75 °C. Our results were similar to other endoglucanases from *Trichoderma viride* HG 623 with temperature and pH of 55 °C with pH 5 and stable for 1 h from 35 to 55 °C between 3.0–7.5 pH (Huang et al. 2013). Cellulase from *Fusarium oxysporum* JRE1 strain exhibited maximum activity at pH 5.6 retaining 75 % activity between pH 4.0–7.0 and 70 % activity around 25–37 °C (Dar et al. 2013).

#### Effect of metal ions, additives, detergents, surfactants, oxidizing agents and organic solvents

The effect of various metal ions on endo β-1,4-d-glucanase activity at different concentrations (1–10 mM) is shown in Table S1 (SI). The metal ions such as Ca^2+^, Mg^2+^, Mn^2+^, Fe^2+^ and Co^2+^ activated the enzyme at 1 mM concentration whereas Hg^2+^, Pb^2+^, Zn^2+^ and Cd^2+^ inhibited the enzyme. Similar results have been reported for endo β-1,4-d-glucanase from *Fusarium oxysporum* JRE1 (Dar et al. 2013) and *Penicillium pinophilum* MS20 (Pol et al. 2012).

Additives like DTT, β-mercaptoethanol, EDTA and urea activated the enzyme and PMSF, *N*-bromosuccinimide, DMSO, iodoacetamide, *p*-CMB and 1, 10-phenathroline inhibited the enzyme as shown in Table S1 (SI). Endo β-1,4-d-glucanase inhibition in the presence of iodoacetamide and *p*-CMB indicate the binding to the –SH groups. Activation by DTT and β-mercaptoethanol indicate the presence of thiol groups at the active sites and these chemicals cause the reduction of disulphide bonds and reactivate the enzyme (Singh et al. 1990). Inhibition by *N*-bromosuccinimide suggests the presence of tryptophan residues in the active sites (Kaur et al. 2015). Similar results have been reported for cellulases from *Bacillus subtilis* YJ1 (Yin et al. 2010) and *Bacillus vallismortis* RG-07 (Gaur and Tiwari 2015).

Endo β-1,4-d-glucanase showed good stability in the presence of detergents retaining 90, 85, 68, 71 and 76 % activity with 1 % of SDS, sodium tetraborate, tide, ariel and surf excel, respectively. Surfactants like tween-20, tween-40, tween-80 and triton X-100 retained greater than 80 % of the activity at 1 %. Endo β-1,4-d-glucanase retained 64, 73 and 40 % of activity with 1 % oxidizing agents like sodium perborate, sodium hypochlorite and hydrogen peroxide, respectively (Table S2, SI). Detergents may cause alterations in the structural and conformational characteristics of enzymes (Bajaj et al. 2009). The detergent stability of cellulase is an important attribute to be an effective additive in commercial detergents. A cold-active endoglucanase from *Aspergillus terreus* strain AKM-F3 was found to be resistant to triton X-100 (Maharana and Ray 2015). Appreciable stability of cellulase from *Bacillus vallismortis* RG-07 for non-ionic surfactants and detergent at 1 % and oxidizing agents at 0.1 % has been reported (Gaur and Tiwari 2015).

The enzyme showed more than 75 % of relative activity in most of the organic solvents like glycerol, ethanol, methanol, acetone, propanol, petroleum ether, isopropanol, benzene, cyclohexane, hexane, butanol and toluene even at 30 % concentration (Table S3, SI). Solvent tolerant enzymes are needed for industrial applications. The enzyme stability in organic solvents may be attributed to its ability to form numerous hydrogen bonds with water, leading to structural flexibility and conformational mobility (Klibanov 2001). In agreement to our results, cold-active endoglucanase from *Aspergillus terreus* strain AKM-F3 and cellulase from *Bacillus vallismortis* RG-07 was also found to be stable in most organic solvents (Gaur and Tiwari 2015; Maharana and Ray 2015).

#### Substrate specificity and enzyme kinetics

Purified endo β-1,4-d-glucanase exhibited highest substrate specificity with CMC (Table S4, SI). It also showed slight activity with filter paper and microcrystalline cellulose. In agreement with our findings, endoglucanse from *P. betulinus, Bacillus subtilis* YJ1 and *Daldinia eschscholzii* also showed highest substrate specificity for CMC (Karnchanatat et al. 2008; Valaskova and Baldrian 2006; Yin et al. 2010). Endoglucanase reported from *Aspergillus nidulans* showed activity with pNP-β-d-lactopyranoside and pNP-cellobioside substrates, and hence belonged to GH7 family (Kaur et al. 2015).

**Fig. 2:**
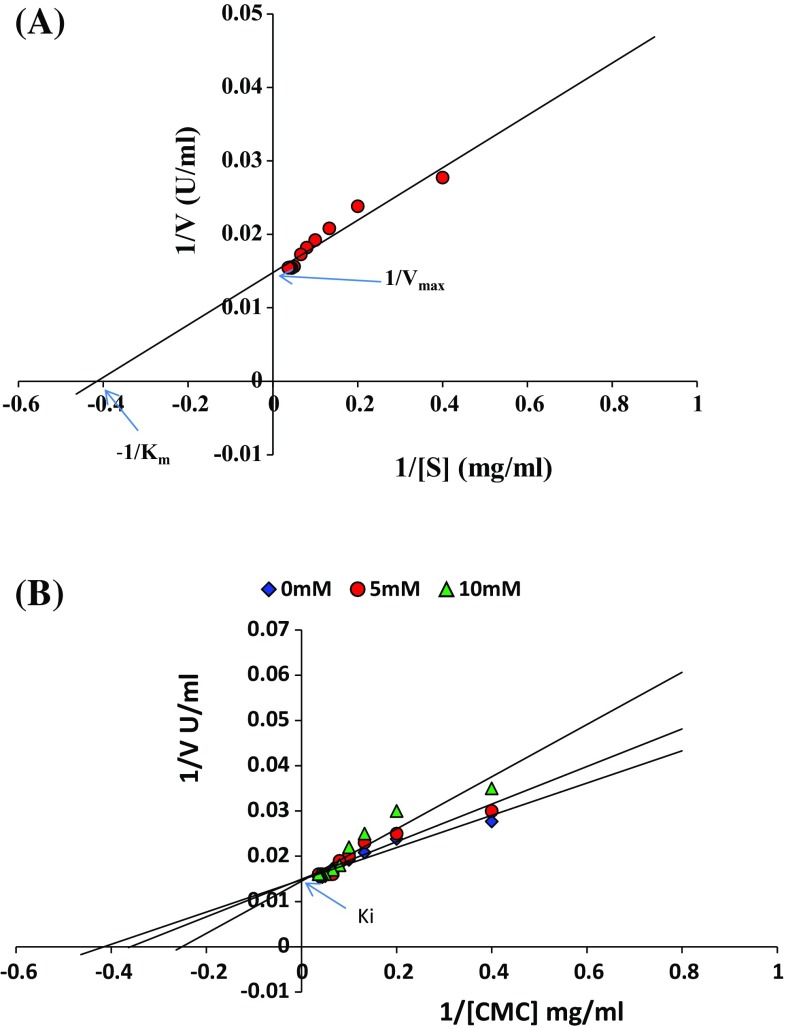
Determination of kinetic parameters *K*_m_ and *V*_max_ by Lineweaver–Burk plot (**a**) and inhibition kinetics by cellobiose on purified endo β-1,4-d-glucanase (**b**)

Furthermore, endo β-1,4-d-glucanase activity increased with increasing substrate concentration up to 22.5 mg/mL, and then saturation was observed due to the saturation of enzyme active sites. Kinetics of enzyme revealed the *K*_m_ and *V*_max_ of 2.5 mg/mL and 83.75 U/mg, respectively, for CMC shown in Fig. 2a. Lower *K*_m_ value indicates strong affinity for the substrate CMC. A *K*_m_ and *V*_max_ of 19.39 g/L and 0.0948 mM/L/min, respectively, was reported by *T. harzianum* IOC-3844 (de Castro et al. 2010). Endoglucanase from *Penicillium pinophilum* MS20 was active towards CMC with *K*_m_ of 4.8 mg/mL and *V*_max_ of 78.5 U/mg (Pol et al. 2012). Inhibition studies showed cellobiose to be competitive inhibitor of endo β-1,4-d-glucanase in the presence of CMC substrate with a *K*_i_ of 0.066 M shown in Fig. 2b. Similar observations were also reported for endoglucanase from *Daldinia eschscholzii* (Karnchanatat et al. 2008). About 87 % of activity was retained by purified endo β-1,4-d-glucanase when compared to crude enzyme with 30 % activity after 60 days of storage stability (Fig. S3, SI).

### Enzymatic hydrolysis and ethanol fermentation

**Fig. 3:**
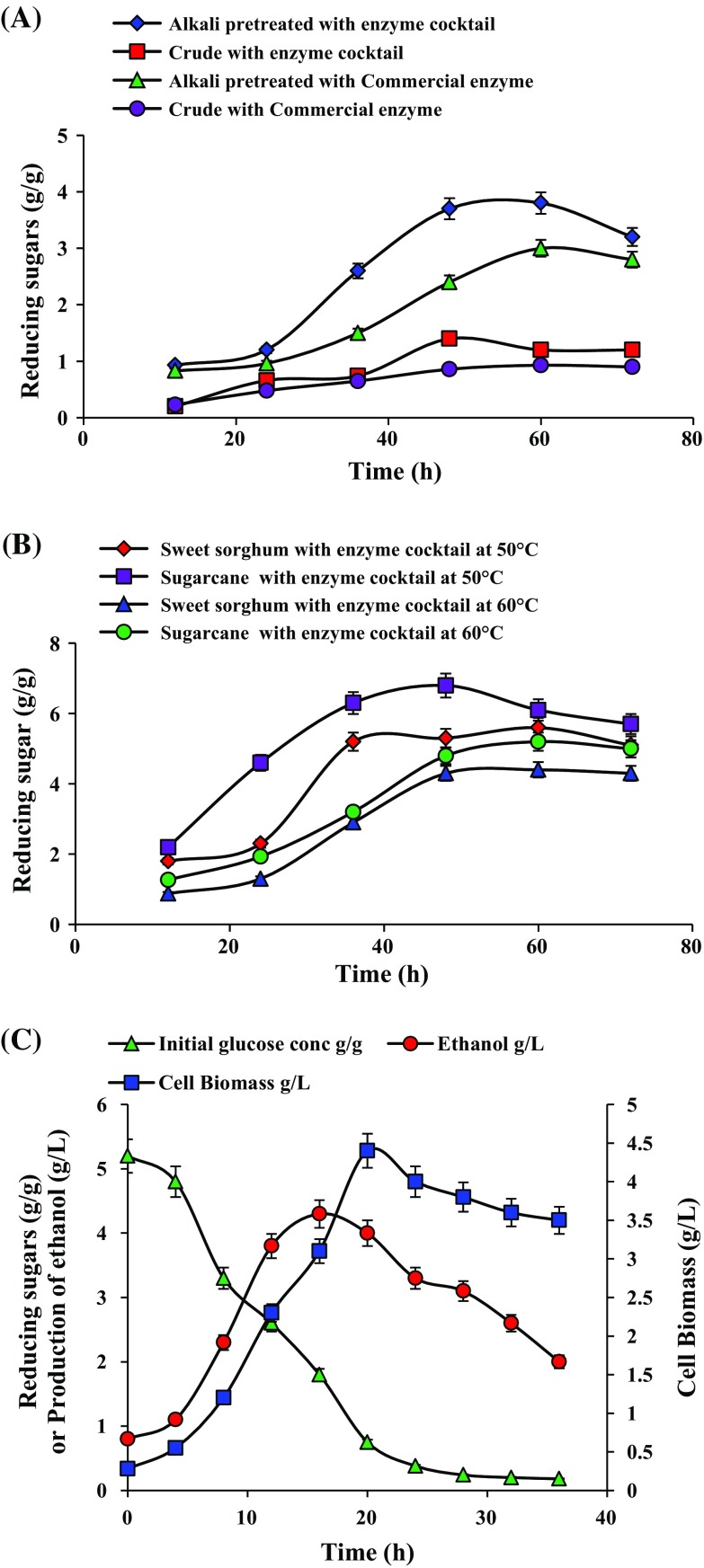
Enzymatic hydrolysis of untreated and pretreated sweet sorghum bagasse with purified endo β-1,4-d-glucanase mixed cocktail and commercial enzyme at 40 °C (**a**), optimization of temperature and time for enzymatic hydrolysis of alkali pretreated sweet sorghum and sugarcane bagasse with purified endo β-1,4-d-glucanase mixed cocktail (**b**), and production of ethanol from sweet sorghum bagasse hydrolyzate fermented by *Saccharomyces cerevisiae* NCIM 3594 (**c**). Data values represent average of triplicates and *error bars* represent standard deviation

In the process of production of cellulosic bioethanol, pretreatment and enzymatic hydrolysis is the crucial steps for saccharification and the reducing sugars could be fermented to ethanol. Untreated and alkali pretreated sweet sorghum bagasse and sugarcane bagasse were used to test the ability of the purified endo β-1,4-d-glucanase with other crude hydrolytic enzyme cocktail and commercial cellulase for the production of fermentable sugar by enzymatic hydrolysis. Alkali pretreated sweet sorghum and sugarcane bagasse released higher amounts of reducing sugars as compared to untreated. Maximum reducing sugars of 3.7 and 5.2 g/g were produced from sweet sorghum and sugarcane bagasse, respectively, at 48 h when treated with purified endo β-1,4-d-glucanase mixture of cocktail, in comparison to commercial cellulase which produced 2.4 and 4.3 g/g of reducing sugars with sweet sorghum and sugarcane bagasse, respectively, at 48 h represented in Fig. 3a. Hence, the efficient bioconversion of lignocellulosic biomass necessarily requires the synergetic action of cellulolytic enzymes, depolymerizing and debranching hemicellulolytic enzymes. Enzymatic hydrolysis of the alkali pretreated sweet sorghum and sugarcane bagasse into glucose was optimized. Optimization of hydrolysis time and temperature revealed high amounts of sugars 5.2 g/g at 36 h and 6.8 g/g at 48 h from alkali pretreated sweet sorghum and sugarcane bagasse, respectively, at 50 °C indicated in Fig. 3b. Therefore, these process parameters play an essential role in hydrolysis of lignocelluloses to get efficient yield of reducing sugars. Moreover, short processing times are a key parameter to an economically viable industrial process. Formulation of cellulase enzymes is challenging for hydrolysis process but will not only lower the enzyme loadings but also reduce the capital cost in a cellulosic bioethanol production. In agreement to our results, crude enzyme cocktail from *T. harzianum* KUC1716 and *S. commune* KUC9397 could replace the commercial enzymes with approx 80 % hydrolysis yield (Lee et al. 2015). A similar study reported by Patel et al. (2015) on hydrolysis of pretreated maize stover suggests that enzyme cocktail with commercial cellulase results in better sugar release. Vimala et al. (2011) studied the effect of different pretreatment strategies for enzymatic hydrolysis of sorghum straw and found that alkali delignified treatment released higher sugars. To minimize the process cost is of great importance for an economical cellulose-based ethanol production. Even if the new enzyme mixes are said to have improved efficiency, the enzyme cost is still contributing to a big part of the total production cost. The present study indicated that the purified endo β-1,4-d-glucanase with cocktail obtained from *Trichoderma harzianum* strain HZN11 enhanced the efficiency of biomass hydrolysis.

In SHF, ethanol produced was qualitatively detected by GC–MS. Spectral analysis for ethanol produced from sweet sorghum bagasse after 24 h by *Saccharomyces cerevisiae* NCIM 3594 showed two peaks in Fig. S4A (SI). Peak 1 showing a retention time of 1.585 with a molecular mass of 58 indicating the presence of acetone according to the data published previously (Tiscione et al. 2011) whereas peak 2 showing a retention time of 1.643 with a mass of 46 indicating the presence of ethanol by comparison with standard ethanol peak in Fig. S4B (SI). Tiscione et al. (2011) also obtained a similar mass spectrum for ethanol by GC–MS indicating this to be an excellent method for ethanol detection by GC-FID and simultaneous confirmation by MS. In SHF, the fermentation of sweet sorghum hydrolyzate by *Saccharomyces cerevisiae* NCIM 3594 produced ethanol (4.3 g/L) at 16 h indicating the utilization of reducing sugars. Reducing sugar concentration in hydrolyzate decrease from 5.2 to 0.38 g/L within 24 h of fermentation with a cell biomass of 4.4 g/L indicated in Fig. 3c. Mukhopadhyay and Chatterjee (2010) reported 4.5 g/L ethanol with pretreated water hyacinth after 72 h of enzymatic hydrolysis in SHF. Sorghum juice and sorghum press cake fermentation produced 9.2 and 5.6 % (w/v) ethanol (Mamma et al. 1995). Interesting one-pot bioethanol production process with *A. cellulolyticus* C-1 and *S. Cerevisiae* co-cultures using Solka-Floc were reported (Park et al. 2012). End product inhibition by glucose is a serious problem in SHF. The main advantage is the possibly to separately optimize the process steps. Hence, we report the successful production of bioethanol by SHF of sweet sorghum bagasse with good ethanol concentration.

### SEM and elemental analysis

**Fig. 4:**
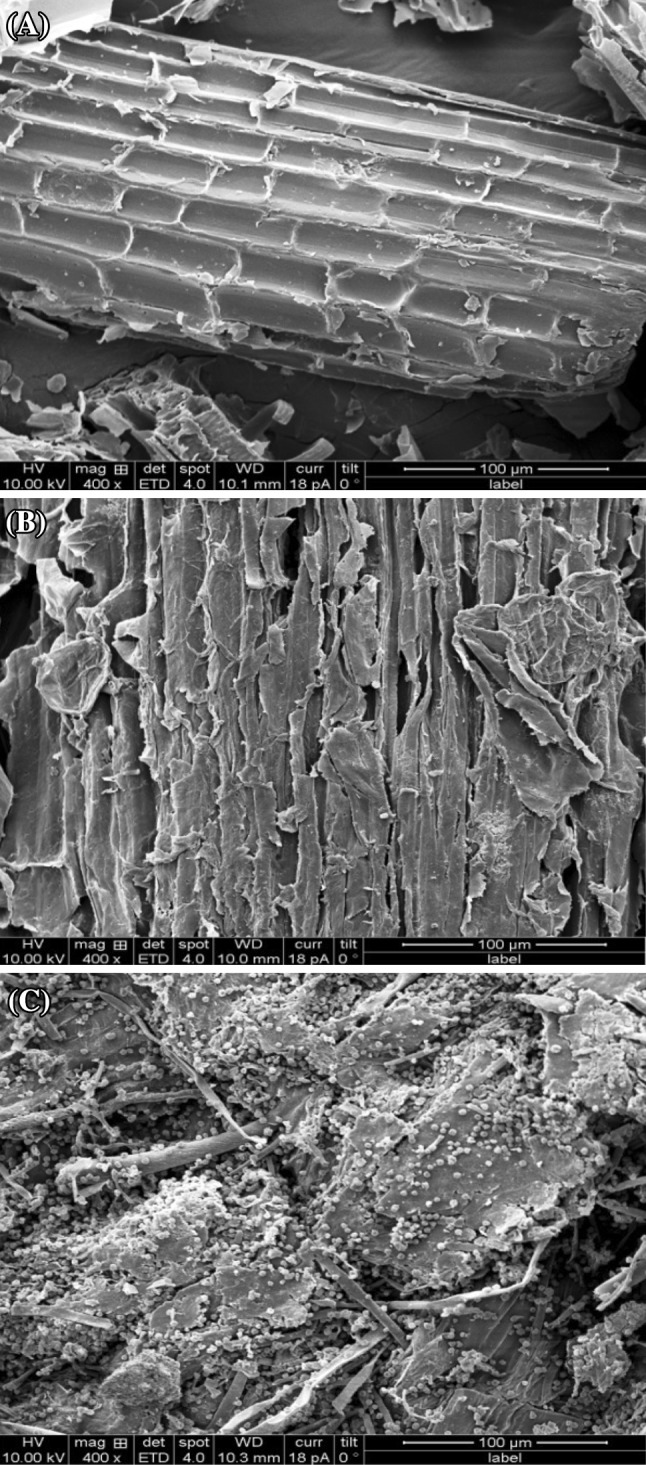
SEM analysis of untreated sweet sorghum bagasse (**a**), alkali pretreated sweet sorghum bagasse (**b**) and pretreated sweet sorghum bagasse hydrolyzed by *Trichoderma harzianum* strain HZN11(**c**)

Scanning electron microscopy studies depict the plant tissue’s morphological and structural changes occurring during pretreatment and hydrolysis. Untreated sweet sorghum bagasse showed a unique structure of the fibers. No pores occurred in large amount as the entire structure was closed indicating to be recalcitrant observed in Fig. 4a. It also reveals intact plant cell wall with vascular bundles and a highly fibrillar structure.SEM image of alkali pretreated sweet sorghum bagasse depicts the alteration in fibrillar structure analyzed in Fig. 4b. Pretreatment usually dislocates the bonding among cellulose, hemicelluloses and lignin by dissolving hemicelluloses, but major microfibrous cellulose structures remain unaltered and some lignin-carbohydrate complexes may be packed on the surface of the cellulose fibers. During pretreatment, hydrocarbons are removed and initiates development of cracks on the lignocellulosic fiber, further increasing the porosity which exposure the cellulosic portion for efficient bioconversion. SEM image of sweet sorghum bagasse hydrolyzed by *Trichoderma harzianum* strain HZN11 showed intact cells on the substrate particles clearly observed in Fig. 4c. Hydrolysis increases the porosity and evidence the utilization of cellulosic material for sugar production. SEM has been also employed to study the surface morphological changes of chars obtained from gasification process of switchgrass, sorghum and red cedar char (Qian et al. 2013). Hard raw sugarcane bagasse and holes after treatment was evidenced indicating saccharification process by SEM analysis (Irfan et al. 2011). A smooth surfaced compact structure and intact morphology of untreated and significant increase in porosity of pretreated sorghum stem was visualized by SEM (Nikzad et al. 2014).

The elemental analysis of biomass was carried out by SEM equipped with EDX technique. Usually raw biomass has high amounts of potassium (K) and chorine (Cl) as they remain in ionic form and are not metabolized by the plant (Lehmann et al. 2011). The SEM/EDX analyses are shown in Fig. S5A (SI) for untreated, Fig. S5B (SI) for alkali pretreated and Fig. S5C (SI) for *Trichoderma harzianum* strain HZN11hydrolyzed sweet sorghum bagasse. The untreated bagasse was composed of carbon (65.47 %) which was gradually reduced to 60.15 % in pretreatment and further reduced to 58.32 % after hydrolysis indicating the utilization of carbon by the organism for enzyme production (Table S5, SI). The nitrogen content in pretreated bagasse may be exposed due to delignification. The presence of sodium may be attributed to alkali pretreatment process. Gradual decrease in the content of minerals and metals in the hydrolyzed bagasse may be due to its utilization by the organism. The increase in nitrogen content as compared to the raw biomass may be predicted due to the stability of nitrogen containing compounds such as heterocyclic aromatic compounds (Cantrell et al. 2012). Fuel characteristics of biomass were studied by this technique. Hence, we report an interesting study on elemental analysis of sweet sorghum bagasse, thereby understanding the insights of nutritional changes and its utilization by *Trichoderma harzianum* strain HZN11during biomass hydrolysis.

### FTIR analysis

The structural changes in untreated, alkali pretreated and *Trichoderma harzianum* strain HZN11 hydrolyzed sweet sorghum bagasse were analyzed by FTIR. The spectrum of untreated, alkali treated and *Trichoderma harzianum* strain HZN11 hydrolyzed sweet sorghum bagasse are shown in the Fig. S6 (SI). Stretching of hydroxyl group increased after alkali pretreatment and hydrolysis of bagasse. The degradation of fibrillar structure of cellulose and lignin is observed after alkaline treatment. A peak of amines N–H stretch is established around 3419 cm^−1^ in untreated and alkali pretreated bagasse and disappeared in hydrolyzed bagasse. Peak around 2915 cm^−1^ in alkali treated bagasse arises from C–H stretching, further suggesting that the hydrolysis process may destroy aliphatic structures in the biomass. Urethane amides stretch was detected around 1734 cm^−1^ range in untreated bagasse. Strong aromatic ring (aromatic lignin) stretch was observed in 1600–1500 cm^−1^ range with aromatic C=C bending in untreated bagasse. Lower band intensity at this wavelength in pretreated and hydrolyzed bagasse signifies delignification effect. In treated and hydrolyzed bagasse peaks around 1315 cm^−1^ are correlated to absorption by C–H and C–O stretching of acetyl group in hemicelluloses. C–O stretching of aryl ethers, and phenolics of lignin-derived compounds and C–O stretching of pyranone rings and guaiacyl monomers around 1250 cm^−1^ and presence of aliphatic amines C–N stretch around 1244 cm^−1^ in untreated bagasse were observed. Glycosidic linkage was predicted in untreated and pretreated bagasse around 1200–1150 cm^−1^. A C–O or C–O–C stretching around 1161 cm^−1^ was observed predicting cellulose and hemicellulose structure and peaks around 1100–1050 cm^−1^ were attributed to β(1-3) polysaccharide in pretreated and hydrolyzed bagasse. A peak found around 898 cm^−1^ was established for cellulose which increases after pretreatment. FTIR spectra illustrated the delignification activities during pretreatment and further degradation of bagasse by enzymatic hydrolysis by *Trichoderma harzianum* strain HZN11. Previous reports on FTIR analysis of lignocellulosic biomass also conclude similar observations (Adapa et al. 2011; Qian et al. 2013). Similar structural changes have been studied in undecayed and decayed lime wood with *T. viride* (Popescu et al. 2010). The effective utilization of sweet sorghum bagasse for enzyme production is clearly evidenced in our study.

## Conclusion

In the present study, the *Trichoderma harzianum* strain HZN11 produced a highly active endo β-1,4-d-glucanase using sweet sorghum bagasse. The purified endo β-1,4-d-glucanase was found to possess the attributes of industrial enzymes like solvent-thermostable-acidophilic nature. The major bottleneck in the biomass to bioethanol production is the high cost of the cellulolytic enzymes. In the present study, attempts to produce bioethanol from sweet sorghum hydrolyzate by SHF were successful. Enzymatic hydrolysis with purified endo β-1,4-d-glucanase mixed with cocktail was efficient in comparison to commercial enzyme. FTIR and SEM/EDX analysis provided the molecular insights of alkali pretreated and hydrolyzed bagasse indicating hydrolysis of plant biomass. The purified enzyme finds huge potential in biofuel and various other industries.

## Electronic supplementary material

Below is the link to the electronic supplementary material.

Supplementary material 1 (DOCX 688 kb)
