# Supplementary material for: Purification and characterization of endo β-1,4-d-glucanase from Trichoderma harzianum strain HZN11 and its application in production of bioethanol from sweet sorghum bagasse
Source: 3 Biotech. 2016 Apr 12;6(1):101. doi: 10.1007/s13205-016-0421-y (PMC4829572; doi:10.1007/s13205-016-0421-y)
Supplement: Supplementary file 1 — Supplementary material 1 (DOCX 688 kb) [file 13205_2016_421_MOESM1_ESM.docx]

**Supplementary Information**

**Purification and Characterization of Endo β-1,4-D-glucanase from *Trichoderma harzianum* Strain HZN11 and its Application in Production of Bioethanol from Sweet Sorghum Bagasse**

Zabin K. Bagewadi, Sikandar I. Mulla and Harichandra Z. Ninnekar^*^

Department of Biochemistry, Karnatak University, Dharwad 580 003, Karnataka, India

Running title

**Purification of endoglucanase from *Trichoderma sp.***

^*^Corresponding author’s address: Dr. H Z. Ninnekar,

Professor,

Department of Biochemistry,

Karnatak University,

Dharwad 580 003, Karnataka, India.

Tel.: +91-0836-2215243; fax: +91-0836-2747884.

Email:[hzninnekar@yahoo.com](mailto:hzninnekar@yahoo.com)

**Supporting Information: 15 Pages, 6 Figures and 5 Tables**

(A)

(B)

**Fig. S1.** Purification of endo β-1, 4-D-glucanase by DEAE-sepharose (A) and Sephadex G-100 (B) chromatography. Data values represent average of triplicates and error bars represent standard deviation.

(A)

(B)

(C)

(D)

**Fig.S2.** Effect of pH (A) pH stability (B) temperature (C) and temperature stability (D) on purified endo β-1, 4-D-glucanase. Data values represent average of triplicates and error bars represent standard deviation.

**Fig.S3.** Storage stability of crude and purified endo β-1, 4-D-glucanase. Data values represent average of triplicates and error bars represent standard deviation.

(A)


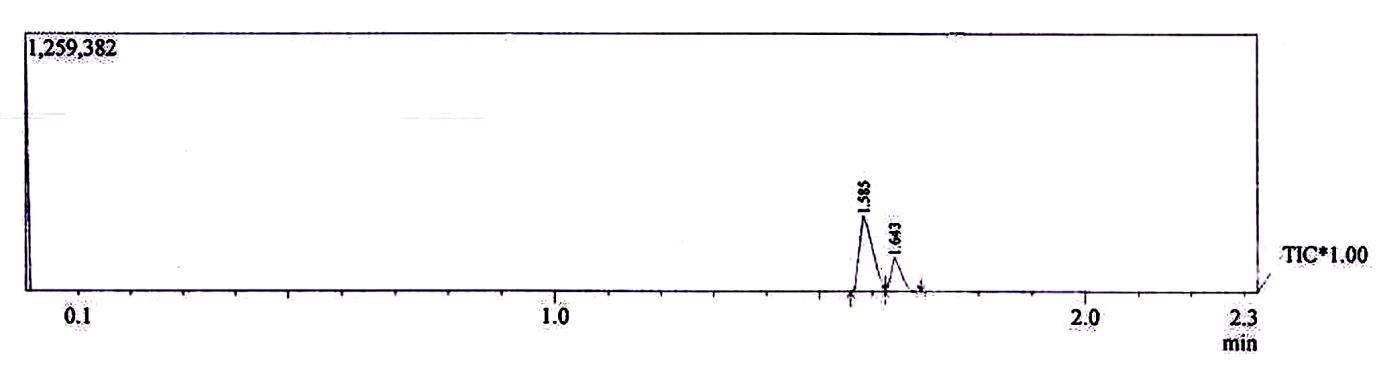


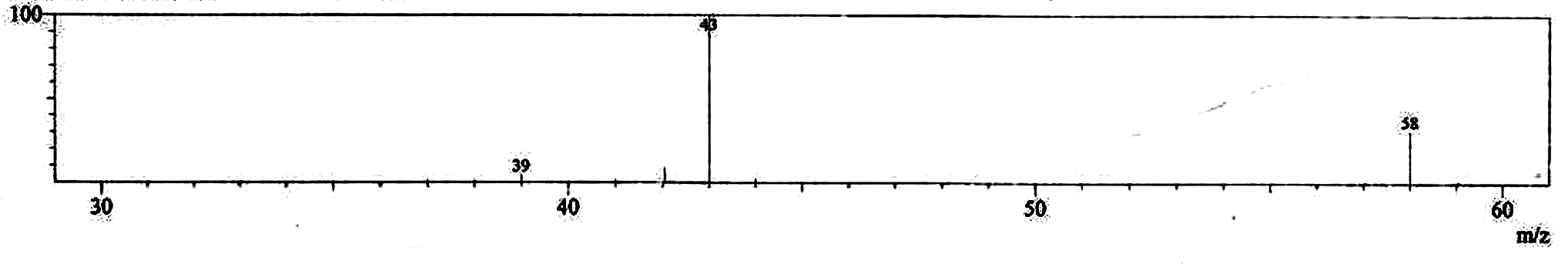

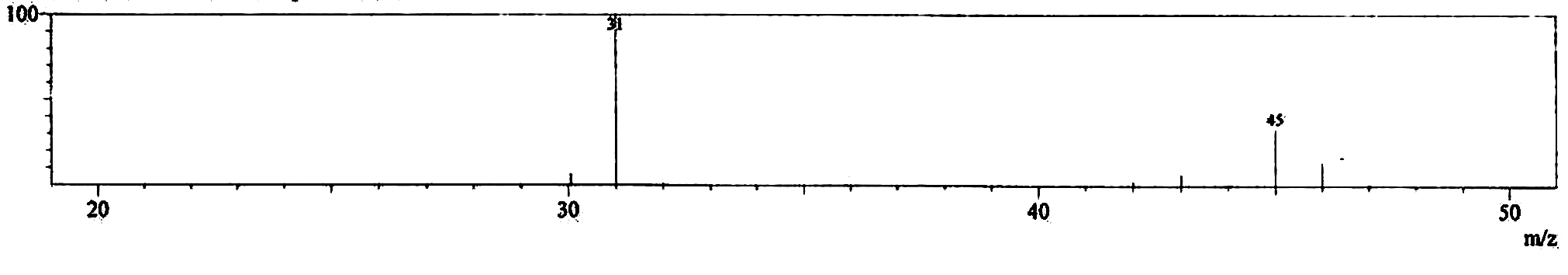


Peak 1

Peak 2

(B)


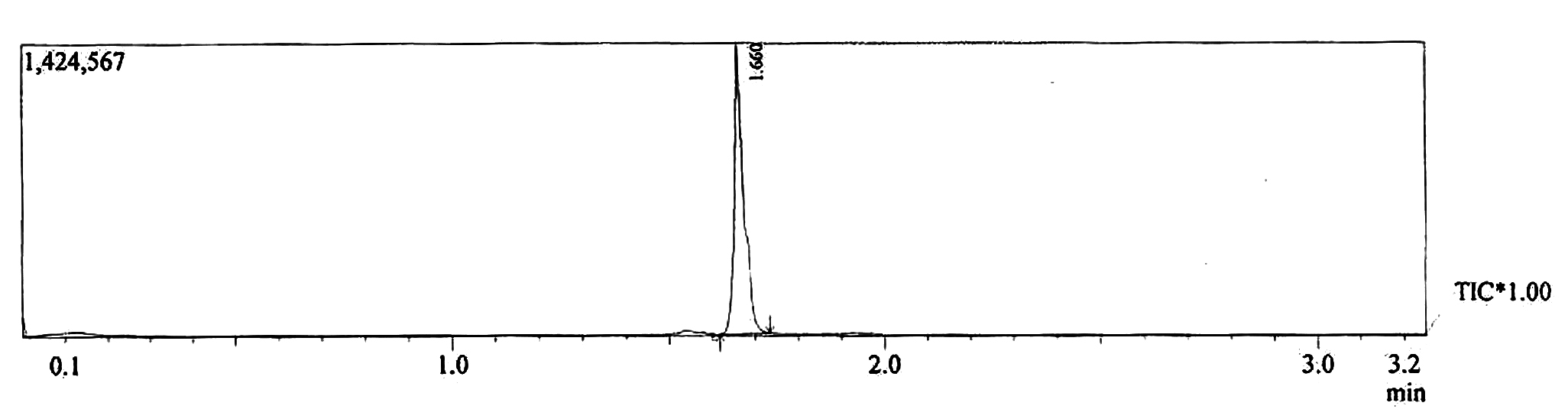


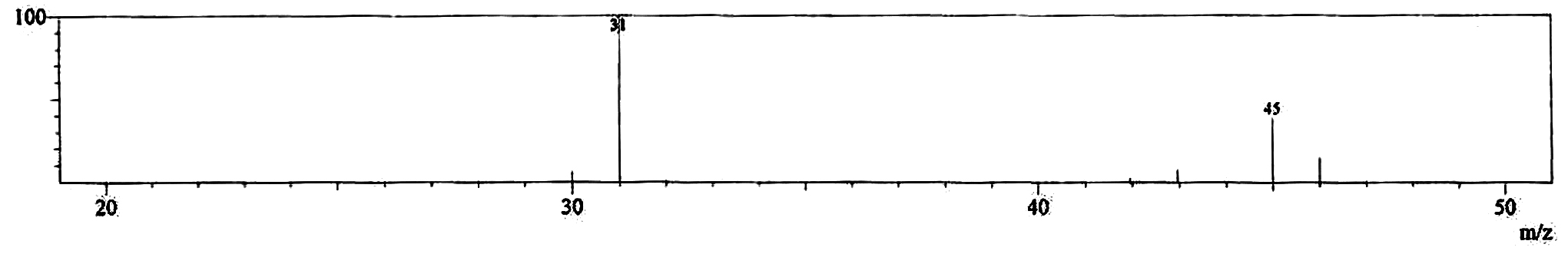


Ethanol

**Fig.S4.** GC-MS spectra of ethanol produced from sweet sorghum hydrolyzate after 24h of fermentation by *Saccharomyces cerevisiae* NCIM 3594 in SHF (A) and GC-MS spectra for ethanol standard (B).

(A)


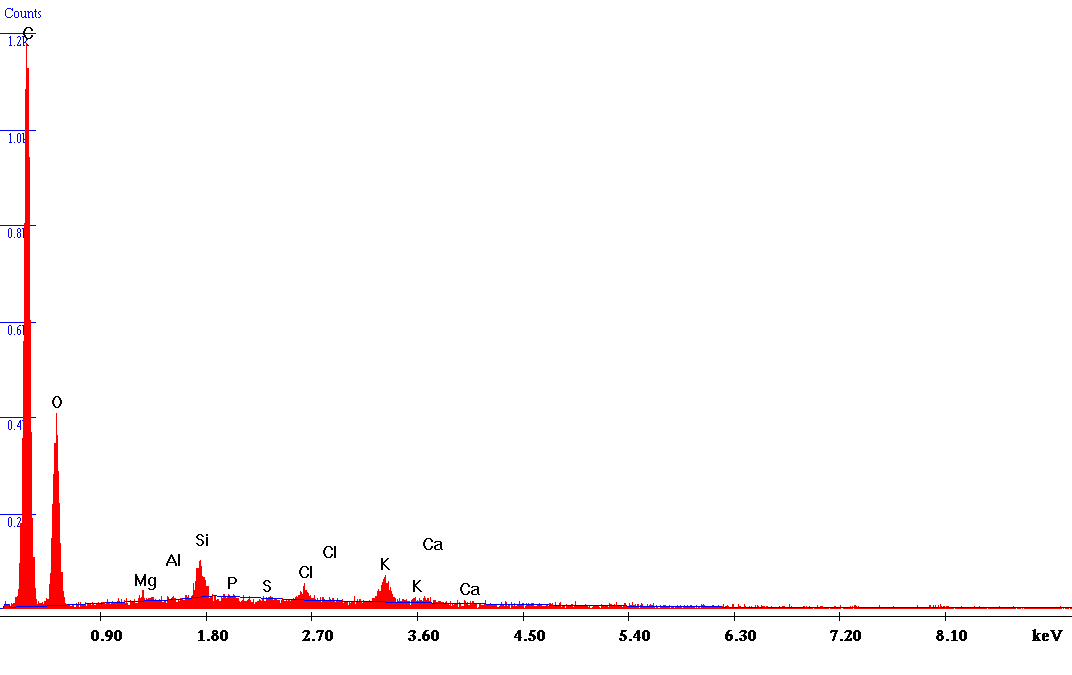


(B)


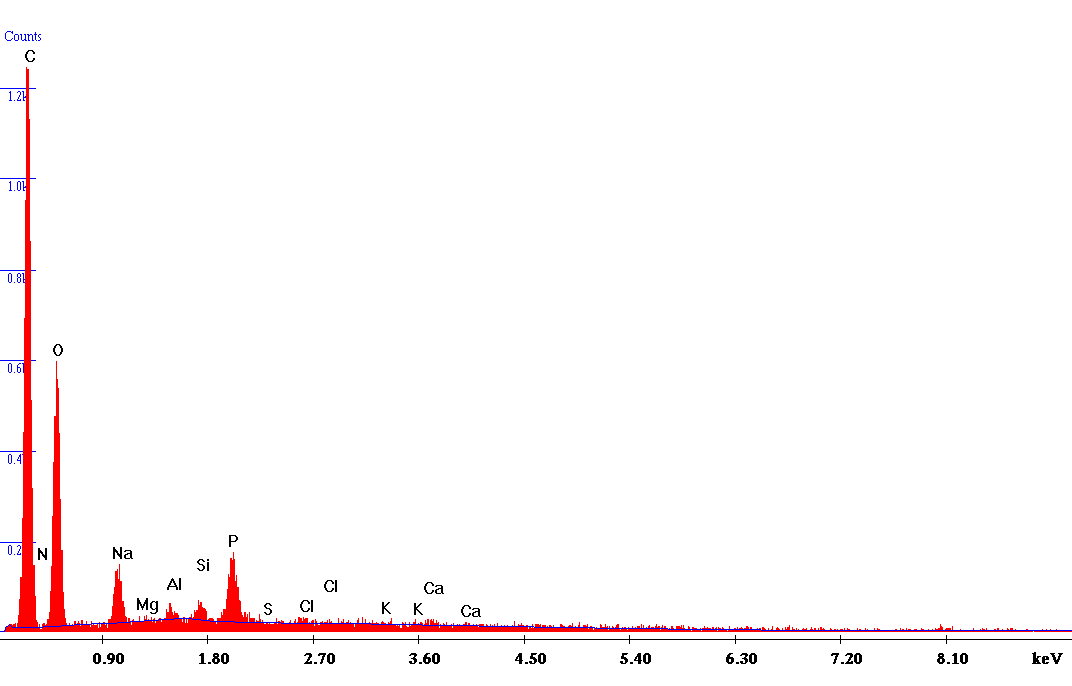


(C)


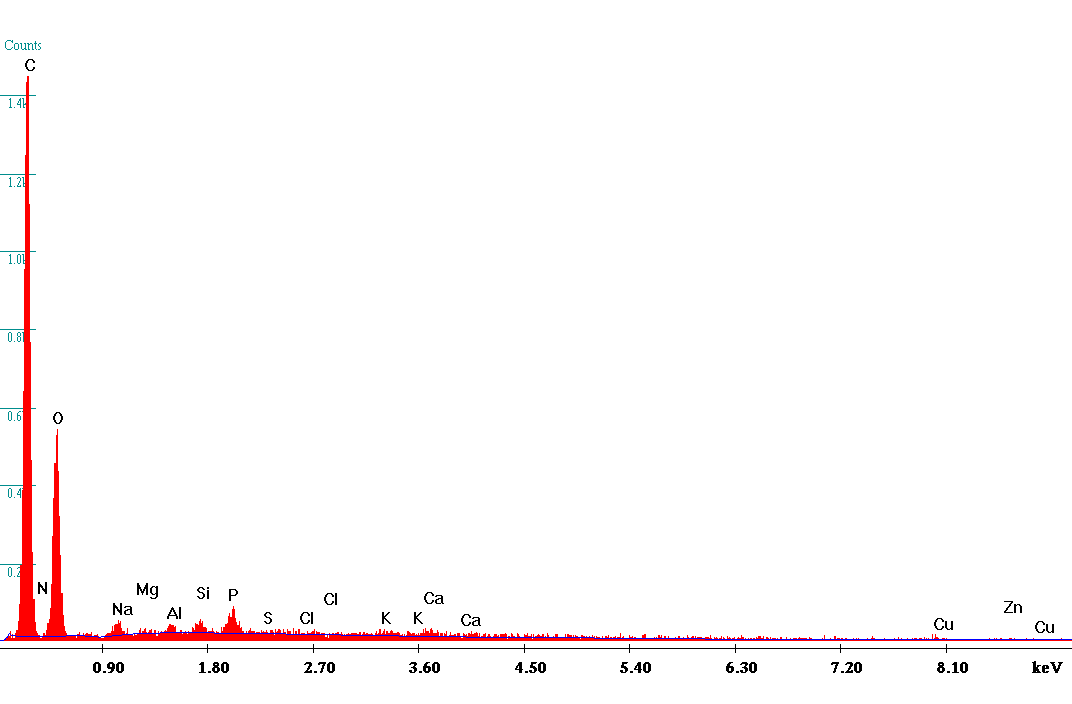


**Fig.S5.** Elemental analysis (EDX) of untreated sweet sorghum bagasse (A), alkali pretreated sweet sorghum bagasse (B) and pretreated sweet sorghum bagasse hydrolyzed by *Trichoderma harzianum* strain HZN11(C).

(A)


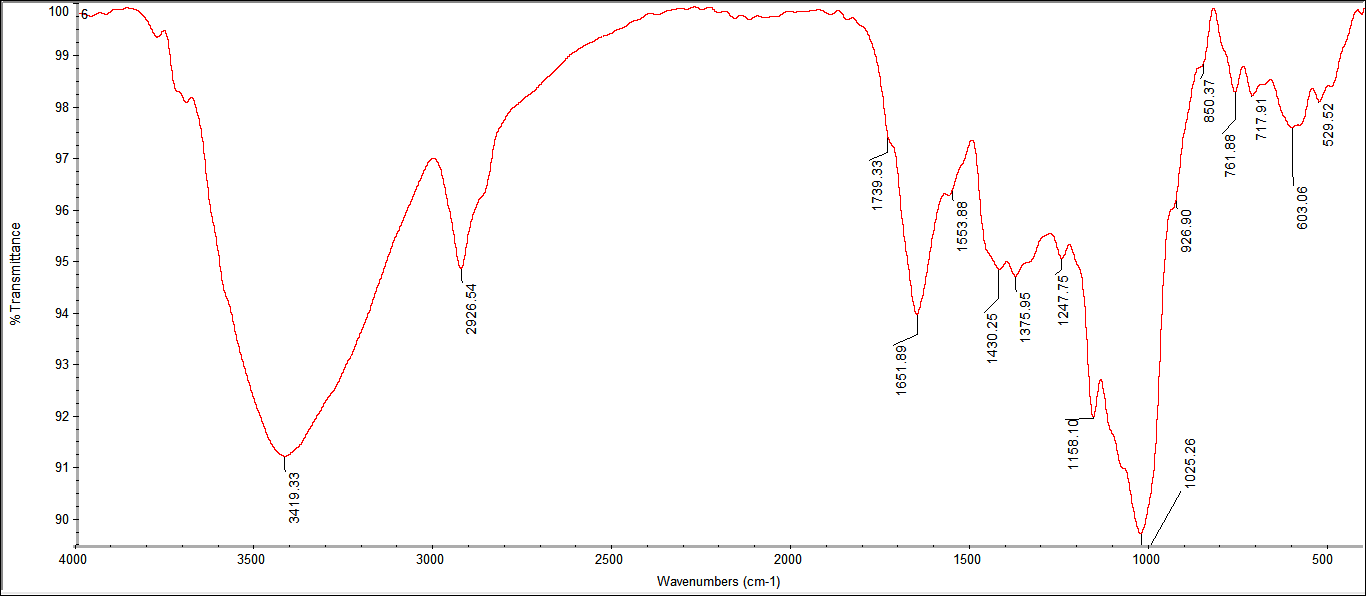


(B)


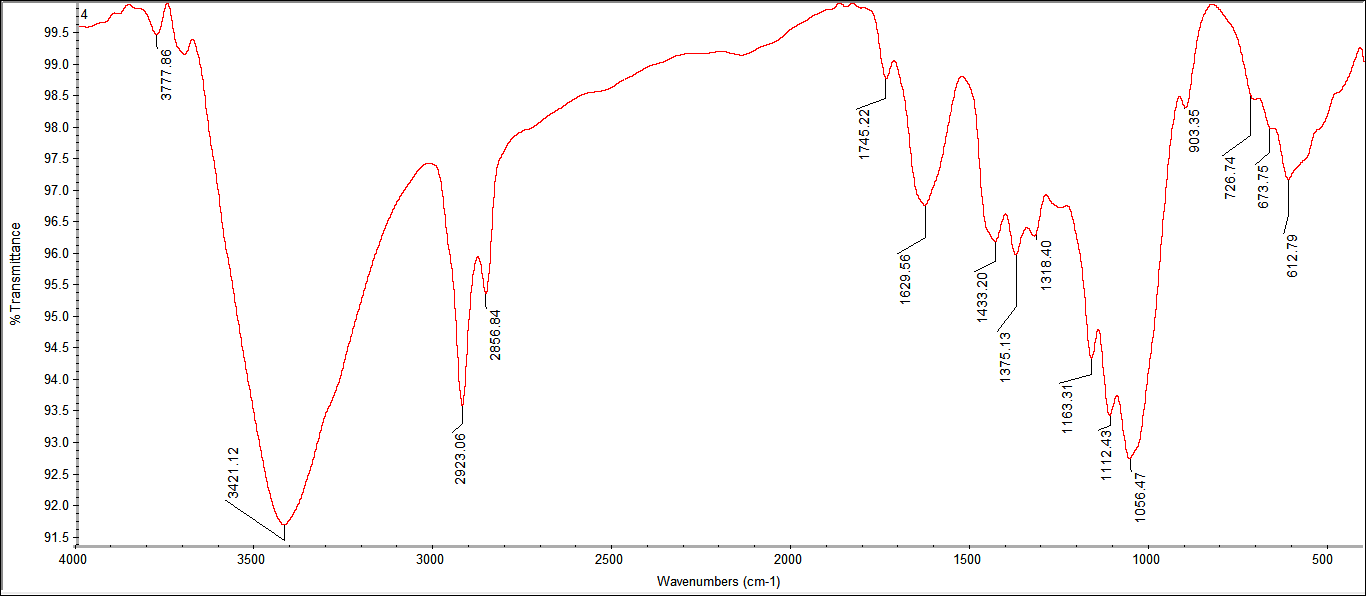


(C)


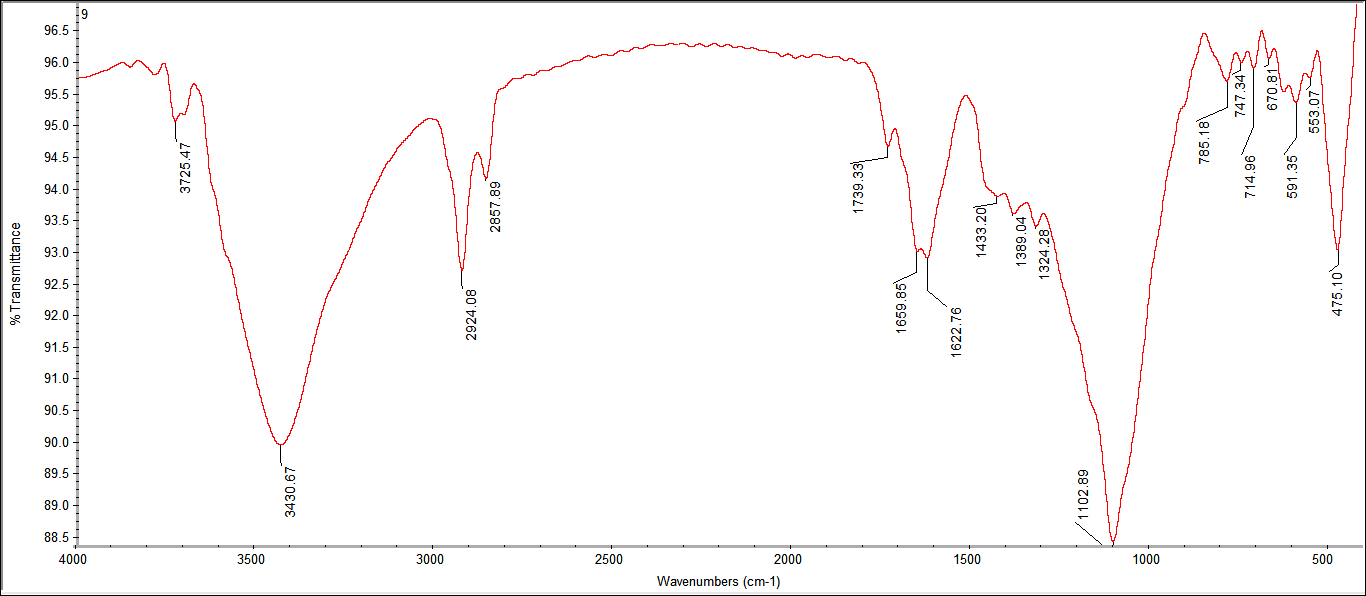


**Fig.S6.** FTIR analysis of untreated sweet sorghum bagasse (A), alkali pretreated sweet sorghum bagasse (B) and pretreated sweet sorghum bagasse hydrolyzed by *Trichoderma harzianum* strain HZN11(C).

Table S1 Effect of different concentrations of various metal ions and additives on purified endo β-1, 4-D-glucanase

| Metal ions and additives | Relative activity (%) | | | |
| --- | --- | --- | --- | --- |
|  | 1 mM | 2 mM | 5 mM | 10 mM |
| Control^a^  Co^2+^  Zn^2+^  Ca^2+^  Mg^2+^  K^+^  Na^+^  Cu^2+^  Hg^2+^  Fe^2+^  Pb^2+^  Ni^2+^  Mn^2+^  Cd^2+^  DTT  β-mercaptoethanol  EDTA  Urea  PMSF  N-bromosuccinimide  DMSO  Iodoacetamide  *p*-CMB  1,10-phenathroline | 100  120  60  130  150  105  80  88  30  110  50  108  110  50  120  118  100  125  70  68  75  72  68  77 | 100  97  55  140  166  95  72  80  18  96  41  96  95  38  117  113  105  120  62  55  68  64  61  72 | 100  85  48  120  172  88  60  68  6  87  34  88  80  20  110  105  100  116  55  42  60  55  52  66 | 100  76  41  80  140  70  50  55  0  72  22  71  72  6  100  102  98  112  46  30  50  41  40  60 |

^a^The activity was assayed in the absence of metal ions or additives was considered as 100%.

Each data value represents average of triplicates.

Table S2 Effect of different concentrations of detergents, surfactants and oxidizing agents on purified endo β-1, 4-D-glucanase

| Detergents, surfactants and oxidizing agents | Relative activity (%) | | |
| --- | --- | --- | --- |
|  | 0.1% | 0.5% | 1% |
| Control^a^  SDS  Sodium tetraborate  Tide  Ariel  Surf Excel  Tween 20  Tween 40  Tween 80  Triton X 100  Sodium perborate  Sodium hypochloride  Hydrogen peroxide | 100  120  98  94  92  93  105  125  115  100  80  90  80 | 100  105  92  80  80  84  90  120  96  92  71  80  65 | 100  90  85  68  71  76  80  105  85  87  64  73  40 |

^a^The activity was assayed in the absence of chemicals was considered as 100%.

Each data value represents average of triplicates.

Table S3 Effect of different concentrations of various organic solvents on purified endo β-1, 4-D-glucanase

| Organic solvents | Relative activity (%) | | |
| --- | --- | --- | --- |
|  | 10% | 20% | 30% |
| Control^a^  Glycerol  Ethanol  Methanol  Acetone  Formic acid  Propanol  Petroleum ether  Isopropanol  Benzene  Cylcohexane  Hexane  Butanol  Toluene | 100  126  98  115  95  70  98  97  100  96  96  92  110  115 | 100  115  94  100  90  61  94  92  97  91  90  85  97  98 | 100  110  90  98  88  44  89  90  92  87  83  76  91  90 |

^a^The activity was assayed in the absence of organic solvents was considered as 100%.

Each data value represents average of triplicates.

Table S4 Substrate specificity of purified endo β-1, 4-D-glucanase

| Substrates | Relative activity (%) |
| --- | --- |
| CMC  Microcrystalline cellulose  Chitin  Cellobiose  Starch  Filter paper  PNP-α-galactopyranoside  PNP-glucopyranoside  PNP-cellobioside  Brichwoodxylan  Oat spelt xylan | 100  40  0  5  0  40  10  12  15  4  3 |

Each data values represent average of triplicates.

Table S5 Element compositional analysis of untreated, pretreated and *Trichoderma harzianum* strain HZN11 hydrolyzed sweet sorghum bagasse by EDX

| Elements | Untreated  Wt (%) | Pretreated  Wt (%) | Hydrolyzed  Wt (%) |
| --- | --- | --- | --- |
| C  N  O  Na  Mg  Al  Si  P  S  Cl  K  Ca | 65.47  0.00  28.51  0.00  0.30  0.10  1.69  0.11  0.08  0.82  2.43  0.49 | 60.15  1.20  30.00  2.79  0.14  0.42  0.74  3.11  0.00  0.27  0.31  0.52 | 58.32  1.51  36.52  1.25  0.14  0.37  0.41  0.87  0.04  0.06  0.20  0.32 |
